# Case Report: Lymphangiogram and embolization for malignant chylothorax in cancer patients

**DOI:** 10.3389/fonc.2025.1586047

**Published:** 2025-05-12

**Authors:** John T. Moon, Hanzhou Li, Omar Abdalla, Nicholas Swilley

**Affiliations:** ^1^ Department of Radiology and Imaging Sciences, Division of Interventional Radiology and Image-Guided Medicine, Emory School of Medicine, Atlanta, GA, United States; ^2^ Vanderbilt University School of Medicine, Nashville, TN, United States

**Keywords:** lymphatic embolization, lymphatic, embolization (therapeutic), coil embolisation, glue embolisation

## Abstract

Tumor-related lymphatic obstruction can cause malignant chylothorax, which can be debilitating. Conventional management includes dietary modifications, percutaneous drainage, and medical management (octreotide), most of which prove refractory in high-output chylothorax cases. Lymphangiogram and embolization in such cases offers a minimally-invasive alternative; however, its use in non-iatrogenic malignant chylothorax is underreported. We present three cases of malignant chylothorax managed with lymphangiogram followed by therapeutic embolization. Case 1: A 70-year-old female with relapsed angioimmunoblastic T-cell lymphoma presents with bilateral chylous effusions refractory to conventional management. Following thoracic duct embolization (TDE) drainage output decreased from over 600 mL/day to less than 200 mL/day, permitting resumption of systemic therapy and subsequent autologous stem cell transplantation. Case 2: A 28-year-old female with ALK-positive non–small cell lung cancer presents with severe respiratory compromise due to extensive mediastinal disease and high-output chylothorax (>1 L/day) refractory to conventional therapy. TDE reduced drainage to less than 150 mL/day, allowing for continued targeted therapy. Case 3: A 70-year-old female with HER2-positive, ER-/PR– breast cancer presents with recurrent right-sided chylothorax despite prior surgical lymphatic ligations. Direct lymphatic leak embolization resulted in marked reduction of chylous output and significant symptom relief. Lymphangiogram with embolization is a safe and effective intervention for malignant chylothorax, regardless of surgical history. Early intervention can alleviate chyle leaks, facilitate ongoing cancer therapy, and improve patient outcomes, making it an important option in multidisciplinary oncology care.

## Highlights

- Novel Application: Lymphangiogram and embolization, traditionally used for postoperative chylothorax, can effectively treat malignant chylothorax arising from tumor-induced lymphatic obstruction.
- Clinical Benefit: Rapid reduction of high-output chyle leaks via lymphangiogram and embolization enables uninterrupted systemic therapy or provides significant symptom relief in advanced malignancies.
- Practice Implication: Early referral for lymphangiogram and embolization in malignant chylothorax may reduce morbidity, shorten hospital stays, and improve overall quality of life in oncology patients.

## Introduction

Chylothorax, the accumulation of chyle in the pleural space, is most commonly associated with traumatic or postoperative injury to the lymphatic channels (1). Malignant chylothorax, however, can result from sequalae of tumor invasion or extrinsic lymphatic obstruction, and is frequently seen in advanced lymphoma, accounting for as much as 70% of cases (2, 3). Chyle leaks can result in severe nutritional deficits, immunosuppression, and significant respiratory distress, all of which can delay oncologic treatment and adversely affect prognosis (4, 5). While initial management includes dietary modifications, medical management with octreotide, and percutaneous drainage, high-output leaks such as those with greater than 1,500 mL/day, are at the threshold for which surgical intervention is recommended.

Lymphangiogram and embolization, including thoracic duct embolization (TDE), is a minimally invasive means by which a lymphatic system is accessed for deployment of coils or liquid embolic agents such as glue. Most commonly utilized in postoperative chylothorax, TDE has demonstrated high technical and clinical success rates in traumatic settings as high as 90% (6). However, its application in malignant chylothorax without recent surgical injury remains less frequently reported, and with variable success rates between 27% and 68% (7–9). The following case series focuses on lymphangiogram-directed embolization performed in three oncology patients with malignant chylothorax, underscoring its potential for rapid symptom relief and support of oncologic care (**Tables 1**, **2**).

**Table 1:** Clinical Characteristics, lymphangiogram and embolization technique, and post-procedural outcomes.

Case	Cancer Type	Chylothorax Cause	Conservative Measures	TDE Technique	Outcome
1	Relapsed AITL	Malignant nodal obstruction	Low-fat diet, octreotide; drainage tube output>600 mL/day	Cisterna chyli access via 22G needle with coil and glue embolization	Chest tube output <200 mL/day; resumed chemotherapy; ASCT achieved
2	ALK-positive NSCLC	Malignant nodal obstruction	Low-fat diet, octreotide; drainage tube output >1 L/day	Cisterna chyli access via 22G needle with coil and glue embolization	Chest tube output <150 mL/day; chest tubes removed; resumed immunotherapy
3	HER2+, ER-/PR– breast cancer	Accessory thoracic duct leak from nodal infiltration	Prior thoracic duct surgical ligation unsuccessful with persistent high output from drainage tube	Direct access via 21G needle with cone-beam CT guidance; sequential glue embolization	Chyle leak resolved; improved respiratory function; palliative benefit achieved

**Table 2:** Event Summary and Complications.

Case	Event Summary	Complication
1	Relapsed AITL diagnosed after worsening dyspnea on exertion → Large pleural effusion diagnosed and confirmed to be chylothorax on fluid studies → TDE performed after lack of improvement with 1 week of low fat diet → Chest tube output decreased over next several days following TDE → Tube was removed after 1 week → Stem cell transplantation 4 months later → PET-CT 3 months after stem cell transplantation confirmed remission.	None
2	Mediastinal mass dx → Airway compromise 2 weeks later treated with tracheal Y-stent, ECMO, pericardiocentesis, and left chest tube placement that day → ECMO decannulated and patient started on Carbotaxol/Pemetrexed the next day → She was started on lorlatinib for ALK+ lung cancer 2 weeks later → After additional 4 days, she decompensated with pericardial and left pleural effusion treated with pericardial window and Pleurx by CT Surgeon → High output chylothorax persisted on low fat diet, resulting in TDE 1 week later and discharged 2 days later → CT surgery removed Pleurx two weeks later → Tracheal stent removed 2 months after TDE.	None
3	Right breast fullness with subsequent 1.3 cm breast mass with node involvement → biopsy diagnosed invasive ductal carcinoma ER-/PR-, HER2+ stage 3A → TCHP started and mastectomy 6 months later → Subsequently completed radiation and started kadcyla which was later changed to Herceptin due to development of thrombocytopenia → 1.5 years after initial diagnosis, she presented with bilateral chylothorax confirmed with high triglycerides in fluid studies → lymphangiogram was initially negative → One month later, patient underwent thoracic duct ligation and right Pleurx catheter placement, with persistent subsequent right side chylothorax → lymphatic embolization performed 1 week later → Chylothorax had resolved within a month after lymphatic embolization.	None

AITL, angioimmunoblastic T-cell lymphoma; TDE, thoracic duct embolization; ECMO, extracorporeal membrane oxygenation; ALK+, anaplastic lymphoma kinase-positive; ER, estrogen receptor; PR, progesterone receptor; HER2, human epidermal growth factor receptor 2; TCHP, docetaxel, carboplatin, trastuzumab, and pertuzumab.The arrows denote the chronological order of events.

## Our standard thoracic duct embolization technique

After obtaining informed consent, the patient is brought to the Interventional Radiology suite, and bilateral groin lymph nodes are accessed with 25 gauge spinal needles with ultrasound guidance. Lipiodol is then injected, and the progression of lipiodol passage is observed under fluoroscopy until visualization of the cisterna chyli. Next, a 22-gauge Chiba needle is advanced through trans-abdominal approach under fluoroscopic guidance. A V18 wire was then advanced through the needle into the thoracic duct. The needle was then exchanged over the wire for a 2.4 French Microcatheter. A small amount of Omnipaque-300 contrast was injected for better opacification of the thoracic duct with visualization of lymphatic leaks where present. Thereafter, a combination of a 5 mm coil is then deployed into the thoracic duct, followed by additional embolization with 1:1 glue:lipiodol mixture. In particularly challenging cases, cone-beam CT guidance allows direct access to the leak site via a 25 gauge spinal needle when a microwire cannot be advanced from the traditional cisterna chyli access.

## Case presentations

### Case 1: Relapsed angioimmunoblastic t-cell lymphoma

**Figure 1:**
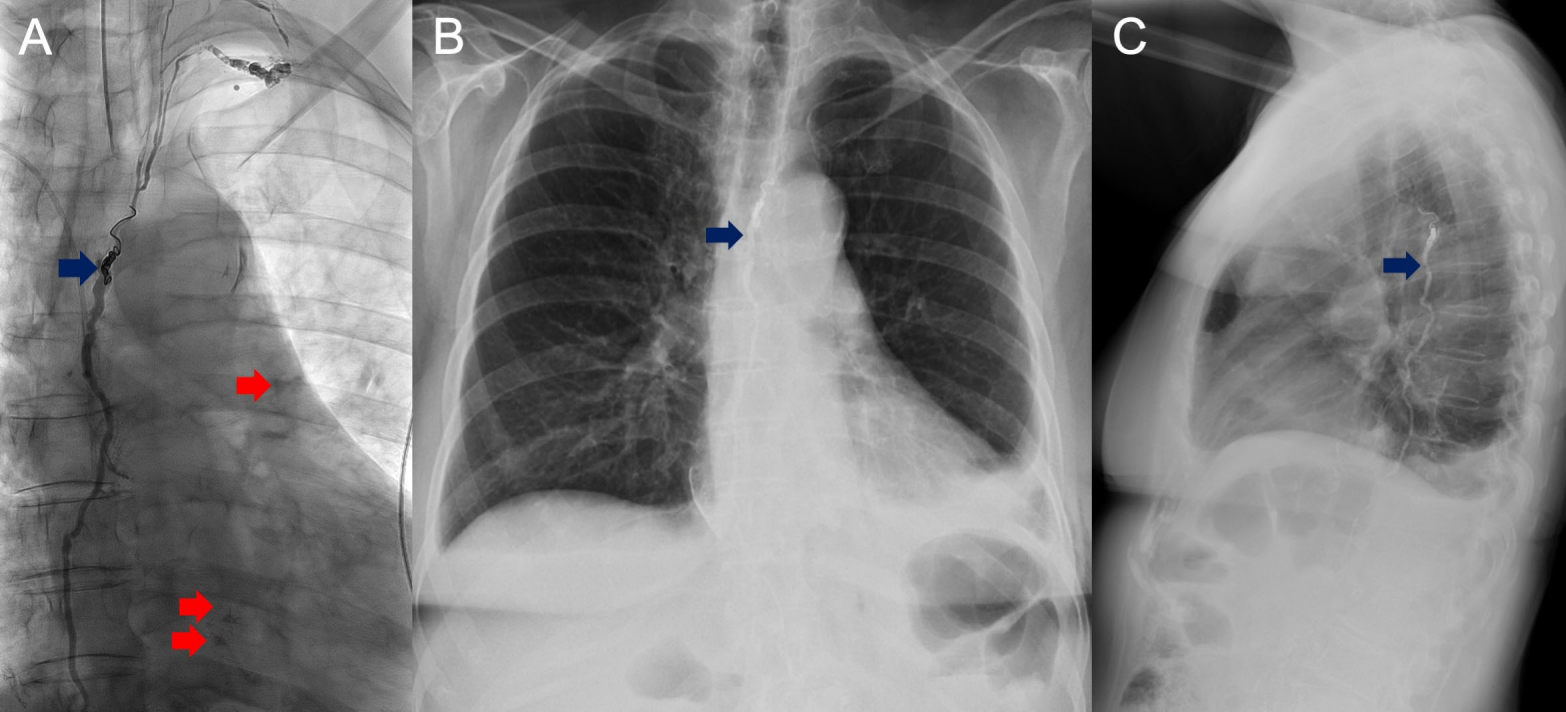
Anteroposterior (AP) orientation fluoroscopic images of Patient 1 with lipiodol-filled thoracic duct with areas of extravasation (red) visualized over the left lower hemithorax status post glue and coil embolization (blue) **(A)**. Removal of left sided pigtail catheter at POD2, and AP **(B)** and lateral **(C)** chest radiograph obtained on POD7 demonstrating residual left sided effusion.

A 70-year-old female with a history of AITL in remission following chemoradiation a decade prior, presents with cervical and supraclavicular lymphadenopathy and constitutional B- symptoms. Imaging demonstrated extensive lymphadenopathy with thoracentesis-confirmed bilateral chylous effusions, and biopsy-confirmed relapse of AITL. Specifically, chylous etiology was confirmed with definitive laboratory evidence: grossly milky yellow appearance, markedly elevated triglyceride levels (801 mg/dL, well above the diagnostic threshold of 110 mg/dL), and lymphocyte-predominant (77%) cellular composition. The patient was placed on a low-fat diet and a left pleural pigtail catheter was placed to drain the left pleural collection. With greater than 600mL of output daily, the decision to proceed with thoracic duct embolization was made with Interventional Radiology as per our standard TDE technique described above with coil and glue embolization (**Figure 1A**). The patient’s output decreased to less than 200 mL daily within 48 hours following 5mm coil and n-butyl cyanoacrylate (NBCA) glue embolization and the pigtail was eventually removed on post-procedural day 2 (**Figures 1B, C**). The patient subsequently resumed chemotherapy and went on to receive autologous stem cell transplantation. She did not develop any complications from the procedure.

### Case 2: ALK-positive non–small cell lung cancer

**Figure 2:**
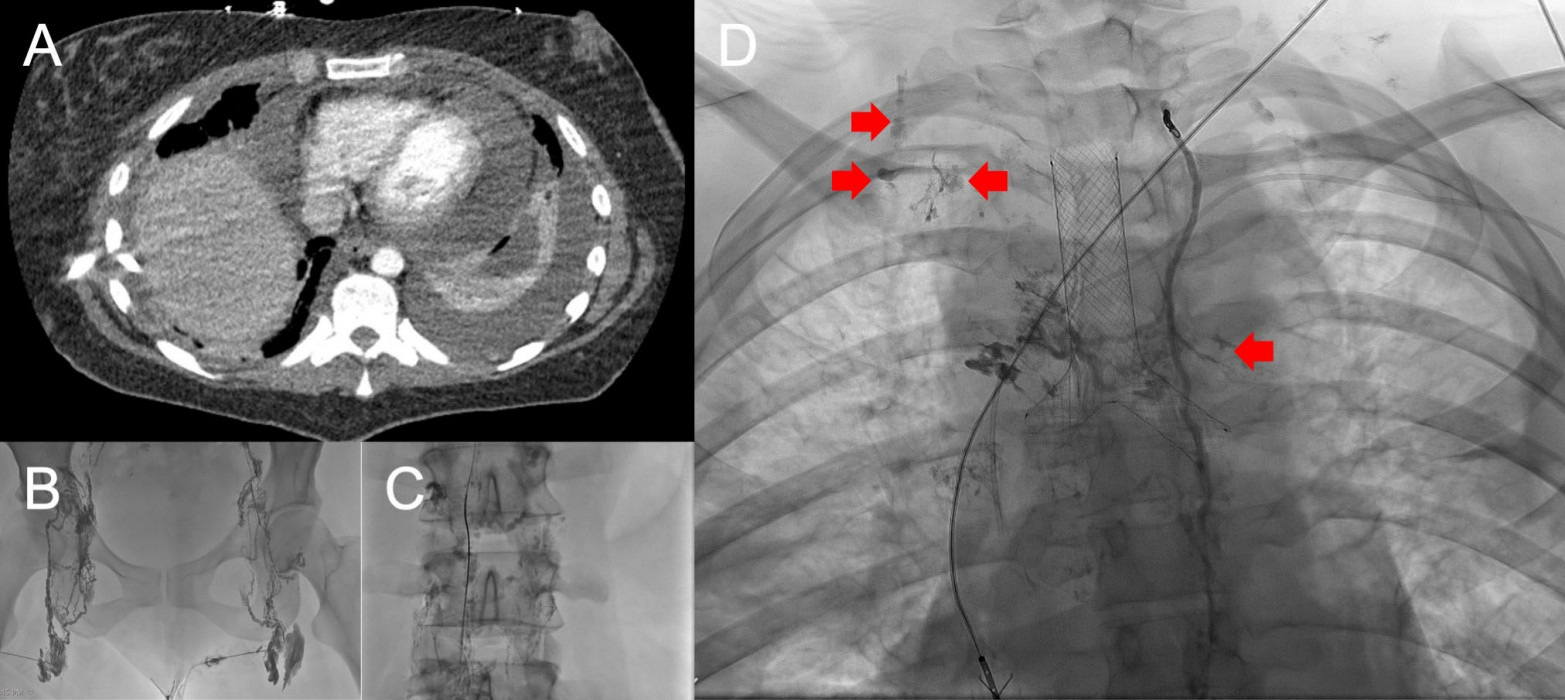
Pre-procedural images of Patient 2 show contrast-enhanced axial CT reformat **(A)** demonstrating large pericardial and moderate left pleural effusion. Intraprocedural AP fluoroscopic images demonstrating difficult groin nodal access **(B)** with eventual microwire and microcatheter crossing the cisterna chylii **(C)** and lymphangiogram demonstrating lipiodol extravasation in the region overlying the right upper and left upper hemithorax **(D)**.

A 28-year-old female with ALK-positive NSCLC initially presents with right neck swelling, cough, and signs of respiratory distress. Subsequent imaging revealed extensive mediastinal lymphadenopathy, pericardial effusion with features of cardiac tamponade, and left sided effusion (**Figure 2A**). Following emergent pericardiocentesis, V-V ECMO support, and placement of a Y-tracheal stent due to severe tracheal narrowing, she was initiated on lorlatinib, but she soon developed bilateral chylothorax with chest tube outputs exceeding 1 L/day. This did not improve with a low-fat diet. With refractory and persistent chylothorax, TDE was performed with 5mm coil and glue (**Figures 2B–D**) following the our institutional TDE technique as described above. Following the procedure, chest tube output decreased to less than <150 mL/day. She did not develop any complications from the procedure.

### Case 3: HER2-positive, ER-/PR– breast cancer

**Figure 3:**
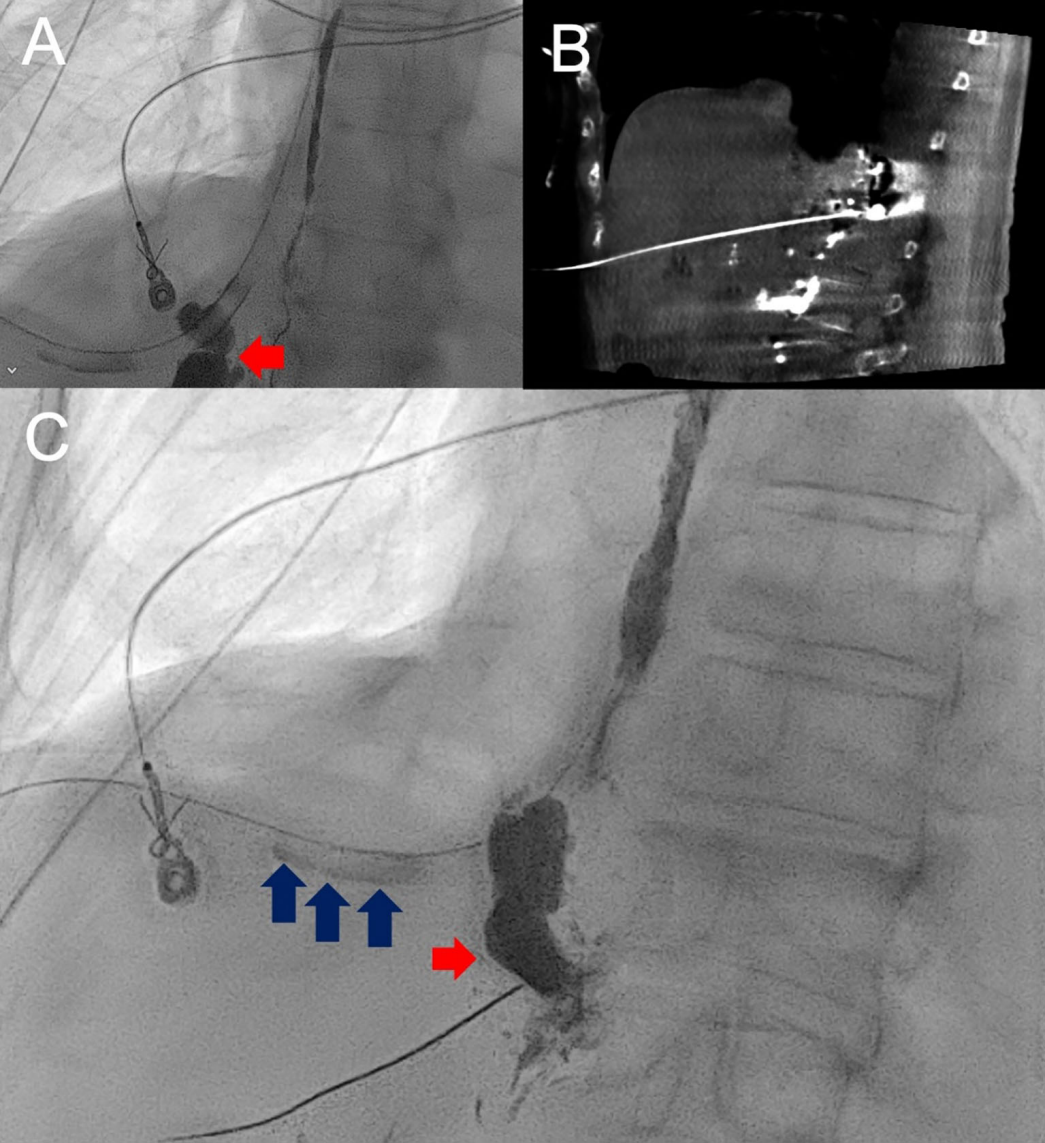
AP fluoroscopic image and lymphangiogram of Patient 3 with lipiodol opacified thoracic duct and extravasation overlying the right lower hemithorax (red arrow) **(A)**. Following unsuccessful catheter and wire manipulation to the known site of leak, cone-beam CT-guided,anterior-approach transhepatic direct stick **(B)** of the leak with subsequent lipiodol injection confirmed leak with visualization of lipiodol passage through the right-sided chest tube (blue arrows) followed by glue embolization **(C)**.

A 70-year-old woman with a history of HER2-positive, ER-/PR– breast cancer status post chemoradiation and bilateral mastectomies, presented for recurrent right-sided chylothorax. Low-fat diet and prior surgical thoracic duct ligation had failed to control the leak, and the patient continued to experience high output. Lymphangiogram did not opacify the thoracic duct, but it identified a leak overlying the right lower hemithorax and subsequent direct access with a 21-gauge x 20 cm long needle and embolization was performed with glue injection (1:1 dilution with lipiodol) through the needle, and confirmed on CBCT. Subsequent chylous output resolved, providing significant palliative benefit for the patient (**Figures 3A–C**). She did not develop any complications from the lymphatic embolization procedure.

## Discussion

Malignant chylothorax can lead to rapid nutritional depletion, immunosuppression, and respiratory compromise that can delay cancer therapy and worsen quality of life for patients. Conventional therapies are often inadequate for high-output chylous effusions. And, while surgical ligation has been the standard for refractory chylothorax, it is often contraindicated in cancer patients due to poor functional status, associated complication rates reported as high as 38.3%, and associated mortality as high as 25% (10, 11). In contrast, lymphangiogram with possible embolization, including thoracic duct embolization, offers a minimally invasive option with lower complication rates (3%) and no reported lymphangiogram-directed embolization-related deaths (7–9). However, in cases of non-iatrogenic chylothorax, accessing the lymphatic system is more technically challenging with as much as 30% of cases precluded secondary to inaccessibility (12).

In this case series, we demonstrate effective management of malignant chylothorax via lymphangiogram-directed embolization. In Case 1, a patient with relapsed AITL achieved a prompt reduction in chyle leak after TDE, which supported patient stabilization for further treatment with chemotherapy and autologous stem cell transplantation. This outcome is critical, as delaying lymphoma treatment may worsen prognosis. In case 2, the patient experienced a significant decrease in chyle output, allowing the continuation of targeted therapy (13). And, in Case 3, lymphangiogram-directed glue embolization proved as an effective therapy for resolving chylous output and improved patient quality of life where surgical ligation was insufficient.

The general technique follows traditional inguinal intranodal injection of lipiodol, followed by needle and microwire access into the cisternal chyli and advancement to the region of leakage (14). Embolization is often accomplished by NBCA glue given its ability to polymerize in the absence of clotting factors in the lymphatic space while the optional addition of coils may be complementary as it can serve as a backstop to prevent glue from advancing into the venous system. Although there have been cases reported where the lymphangiogram alone was therapeutic, our practice cannot attest to this phenomenon (8). Although no immediate complications were not observed in this cohort of patients, the most challenging part of the procedure is getting access to the lymphatic systems and complications can arise from access site bleeding or peritonitis (15). Additionally, embolization of glue into the pulmonary artery has been a reported complication and the intent of the addition of coiling serves to mitigate the risk of glue advancement. Regarding access, our approach consistently utilizes bilateral inguinal lymph node access for initial lymphangiography, followed by direct cisterna chyli puncture for thoracic duct catheterization while alternative approaches via the subclavian vein have been described.

Our case series underscores the importance of early referral to interventional radiology for lymphangiogram with possible embolization when conservative measures fail, that is when there is persistent high output chylothorax over several days. With early intervention, we may offer patients a shortened course of nutritional deficiency, symptomatic respiratory distress in those without indwelling tubes, and stabilization for continued cancer-related treatments.

The novelty of our case series lies in its focus on malignant chylothorax unrelated to surgical injury, an area with limited prior reports. By demonstrating that lymphangiogram-directed embolization can be effectively used across different oncologic contexts (lymphoma, lung cancer, and breast cancer), we add to the growing evidence that minimally invasive management can transform the care of such patients.

## Conclusion

Lymphangiogram-directed embolization is a safe, minimally invasive, and effective treatment option for patients with malignant chylothorax. The above case series demonstrates the clinical and palliative benefit that lymphangiogram-directed embolization can offer for non-iatrogenic etiologies of malignant chylothorax by not only decreasing chylous output but improving nutritional status and clinical condition to be permissive of continued cancer treatments.

## Data Availability

The original contributions presented in the study are included in the article/supplementary material. Further inquiries can be directed to the corresponding author/s.
